# Violence Against Healthcare Workers by Mentally Ill Patients: Impact on Staff in Dementia Wards at Julian Hospital, Norwich

**DOI:** 10.1192/bjo.2025.10379

**Published:** 2025-06-20

**Authors:** Ruzaika Jaufer, Amal Baby, Minfas Affan

**Affiliations:** 1 Norfolk and Suffolk NHS Foundation Trust, Norwich, United Kingdom; 2 St Helens and Knowsley Teaching Hospital NHS Foundation Trust, Norwich, United Kingdom

## Abstract

**Aims:** This study aims to explore the risk factors, impact, and support systems related to violence against healthcare staff by mentally ill patients. It examines the psychological, physical, and professional effects on staff, while assessing the effectiveness of current reporting mechanisms. Addressing the global issue of underreporting violence against healthcare staff, the study seeks to raise awareness, promote safety, and advocate for stronger preventative measures.

**Methods:** This study surveyed healthcare staff across three dementia wards at Julian Hospital in Norwich, using both online and paper questionnaires.

**Results:** The study received 38 responses. The majority were women (23), with 15 men. A large portion of the workforce was younger, with 20 aged 20–35, 15 aged 35–50, and only 3 over 50. Racially, most were Caucasian (21), followed by African (9), Asian (5), and 3 categorized as “other”. Clinical support workers made up 31% of respondents, highlighting their frequent patient interaction. These patterns suggest that younger, female, and front-line staff, particularly clinical support workers, are more vulnerable to workplace violence.

The study found high prevalence of verbal aggression, with 55% of respondents experiencing it almost daily, 21% 2–3 times weekly, and 10% weekly. Physical violence was also significant, with 21% facing it nearly every day and 23% 2–3 times weekly.

The emotional and psychological impact varied among staff. While 45% reported no significant emotional effect, 40% experienced reduced motivation, 10% considered changing jobs, and 5% contemplated changing careers. In terms of mental health and wellbeing, 44% had no effect, 40% reported mild and 16% reported severe psychological strain.

Regarding reporting and organizational support, 75% of participants found the reporting procedure effective, though 92% agreed it was time-consuming. After incidents, 67% felt supported, while 33% did not. Most (75%) felt organizational training provided to handle violent or aggressive situations was adequate, though 25% disagreed. These results highlight areas for improvement, particularly in streamlining reporting and enhancing post-incident support.

**Conclusion:** The study highlights the prevalence and impact of workplace violence on healthcare professionals in mental health hospitals, with younger, female, and front-line staff being most vulnerable. Verbal aggression was reported daily by many, with physical violence also frequent. The impact varied, with some showing resilience, while others faced emotional distress and psychological effects. Although reporting mechanisms and training were generally effective, their time-consuming nature and gaps in post-incident support point to areas for improvement. Addressing these issues with targeted interventions and enhanced support systems is crucial for ensuring the safety and well-being of healthcare workers.

